# Modulares Herzrhythmusmanagement: EMPOWER™ Leadless Pacemaker als Add-on zum EMBLEM™ S-ICD

**DOI:** 10.1007/s00399-025-01113-6

**Published:** 2025-10-29

**Authors:** Boris Rudic, Daniel Dürschmied, Ibrahim Akin, Jürgen Kuschyk

**Affiliations:** 1 I. Medizinische Klinik – Kardiologie, Angiologie, Intensivmedizin, Universitätsmedizin Mannheim, Theodor-Kutzer-Ufer 1, 68167 Mannheim, Deutschland; 2 Deutsches Zentrum für Herz-Kreislauf-Forschung (DZHK), Ruprecht-Karls-Universität Heidelberg, Medizinische Fakultät Mannheim, Mannheim, Deutschland

**Keywords:** Herzrhythmusstörung, Antibradykarde Stimulation, Subkutaner implantierbarer Kardioverter-Defibrillator, Antitachykarde Stimulation (ATP), Ventrikuläre Arrhythmie, Cardiac arrhythmia, Antibradycardia pacing, Subcutaneous implantable cardioverter-defibrillator, Antitachycardia pacing (ATP), Ventricular arrhythmia

## Abstract

Technologische Fortschritte in der Medizin ermöglichen inzwischen die kabellose antibradykarde Myokardstimulation. Kabellose Herzschrittmacher (Leadless Pacemaker, LP) wurden bereits von den Firmen Abbott (Abbott Park, IL, USA; Nanostim™, Aveir™ und Medtronic (Minneapolis, MN, USA; Micra AV/VR™) und zuletzt auch von Boston Scientific (Marlborough, MA, USA; EMPOWER™) vorgestellt. Obwohl bereits Kasuistiken mit unterschiedlichen LP in Kombination mit dem EMBLEM™ S‑ICD (subkutaner implantierbarer Kardioverter-Defibrillator) publiziert wurden, besteht nur mit EMPOWER™ LP die technische Voraussetzung zum modularen Herzrhythmusmanagement („modular cardiac rhythm management“, mCRM), bei dem im Bedarfsfall ein ATP-Befehl (Befehl zur antitachykarden Stimulation) vom S‑ICD an den LP gesendet werden kann, wodurch möglicherweise S‑ICD-Schocks reduziert werden können. Zudem kann auch längerfristig eine antibradykarde Stimulation sichergestellt werden. Nach extensiven präklinischen Studien stehen nun die 6‑Monats-Daten der ersten prospektiven multizentrischen Studie (MODULAR-ATP) zur Verfügung, welche die Sicherheit, Leistungsfähigkeit und den Bedarf des mCRM-Systems analysiert hat.

## Abstract (en)

Technological advances in medicine now enable wireless antibradycardia myocardial stimulation. Leadless pacemakers (LP) have already been introduced by Abbott (Abbott Park, IL, USA; Nanostim™, Aveir™), Medtronic (Minneapolis, MN, USA; Micra AV/VR™), and most recently by Boston Scientific (Marlborough, MA, USA; EMPOWER™). Although case reports have been published using different LP in combination with the EMBLEM™ S‑ICD, only the EMPOWER™ LP provides the technological prerequisites for modular cardiac rhythm management (mCRM). In this modular system, an ATP command can be transmitted from the S‑ICD to the LP when required, potentially reducing the number of S‑ICD shocks. Additionally, long-term antibradycardia pacing can be reliably ensured. Following extensive preclinical studies, the 6‑month results of the first prospective multicenter study (MODULAR-ATP) are now available, analyzing the safety, performance, and necessity of the mCRM system.

## Konzept und technische Grundlagen

Das modulare Herzrhythmusmanagement (mCRM) ist eine innovative Strategie zur Behandlung von Herzrhythmusstörungen, die durch eine flexible Kombination verschiedener, drahtlos miteinander kommunizierender Implantate realisiert wird. Im Gegensatz zu traditionellen Systemen mit transvenösen Elektroden bietet das modulare Konzept der Firma Boston Scientific die Möglichkeit, spezifische Therapiekomponenten wie den EMPOWER™ Leadless Pacemaker (LP) und den EMBLEM™ S‑ICD (subkutaner implantierbarer Kardioverter-Defibrillator) individuell auf die Bedürfnisse der Patienten abzustimmen.

Klinisch besteht ein unverändert hoher Bedarf für solche alternativen Ansätze, da konventionelle transvenöse Systeme mit signifikanten Komplikationsraten assoziiert sind, darunter Elektrodenbrüche, Infektionen und Venenverschlüsse [9]. Diese Komplikationen können schwerwiegende klinische Folgen haben, einschließlich notwendiger Revisionseingriffe und erhöhter Morbidität [6]. Das modulare Konzept adressiert diese Herausforderungen durch den Verzicht auf transvenöse Elektroden und ermöglicht gleichzeitig eine personalisierte und bedarfsgerechte Therapieanpassung.

**Abb. 1:**
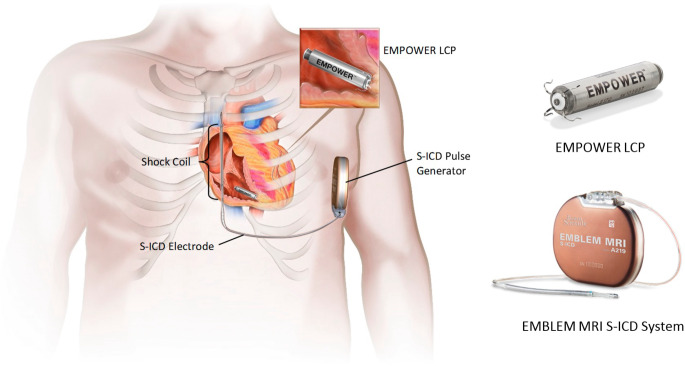
Komponenten des modularen Herzrhythmusmanagement(mCRM)-Systems, bestehend aus dem EMPOWER™ Leadless Pacemaker (LP) im Apex des rechten Ventrikels und dem EMBLEM™ S‑ICD (subkutaner implantierbarer Kardioverter-Defibrillator). (Bild mit freundlicher Genehmigung von Boston Scientific. © 2025 Boston Scientific Corporation oder verbundene Unternehmen. Alle Rechte vorbehalten)

Ein zentraler Bestandteil des mCRM-Systems ist der EMPOWER™ LP, der speziell entwickelt wurde, um mit dem EMBLEM™ S‑ICD drahtlos zu kommunizieren und dadurch eine koordinierte Therapie, insbesondere antitachykarde Stimulation während monomorpher ventrikulärer Tachykardien (VT), aber auch längerfristig eine bedarfsgerechte antibradykarde Therapie zu ermöglichen (Abb. 1). Diese technologische Innovation eröffnet neue therapeutische Möglichkeiten, insbesondere für Patienten, die sowohl von der subkutanen Defibrillation als auch von der antitachykarden Stimulation profitieren könnten, ohne den Risiken transvenöser Sonden ausgesetzt zu sein.

Ziel dieses Übersichtsartikels ist es, das Konzept des mCRM vorzustellen, den klinischen Bedarf herauszustellen und aufzuzeigen, wie präklinische und klinische Studien diese neue Technologie validieren. Darüber hinaus sollen zukünftige Perspektiven und mögliche klinische Implikationen des mCRM-Konzepts diskutiert werden.

## Präklinische Evidenz: Validierung der Systemkommunikation, ATP-Fähigkeit und Explantierbarkeit im Tiermodell

**Abb. 2:**
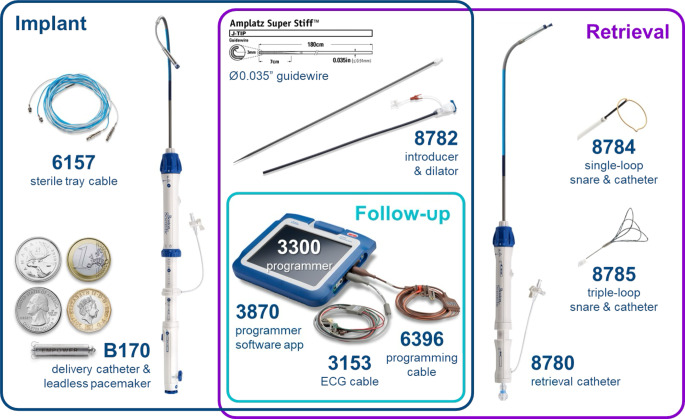
Komponenten für Implantation, Nachsorge und Explantation des EMPOWER™-Leadless-Pacemaker-Systems. *Links* Komponenten für die Implantation, bestehend aus dem B170 Einführkatheter mit integriertem LP, einem sterilen Tray-Kabel (6157) sowie Größenvergleich mit Münzen. *Mitte oben* Zubehörteile wie ein 0,035-Zoll-Amplatz Super Stiff™-Führungsdraht und ein Einführsystem mit Dilatator (8782). *Mitte unten* („Follow-up“): Nachsorgegeräte bestehend aus dem 3300 Programmer, der zugehörigen Programmiersoftware (3870), einem EKG-Kabel (3153) sowie dem Programmierkabel (6396). *Rechts* Explantationskomponenten, darunter der 8780 Retrieval-Katheter, ein Einzelschlingen-Snarekatheter (8784) und ein Dreischlingen-Snarekatheter (8785). (Bild mit freundlicher Genehmigung von Boston Scientific. © 2025 Boston Scientific Corporation oder verbundene Unternehmen. Alle Rechte vorbehalten)

Die präklinische Validierung des mCRM wurde umfassend in mehreren Tiermodellen untersucht. Zentrale Aspekte dieser Untersuchungen waren die langfristige Kommunikationsstabilität zwischen den beiden Geräten, die Effizienz der antitachykarden Stimulation sowie die allgemeine elektrische Performance des LP. Abb. 2 zeigt die Komponenten für Implantation, Nachsorge und Explantation des LP.

Die erste publizierte Proof-of-Concept-Studie erfolgte 2016 in einem Tiermodell an Schafen [15]. In dieser Studie wurde die kombinierte Implantation eines zur antitachykarden Stimulation (ATP) fähigen LP und S‑ICD untersucht. Dabei wurde eine drahtlose, intrakorporale, unidirektionale Gerätekommunikation (S-ICD zu LP) sowie die ATP-Bereitstellung durch den LP getestet. Dazu ist der LP so konzipiert, dass er eine Abfolge kurzer elektrischer Impulse erkennt, die vom S‑ICD gesendet werden. Diese Signale werden von der S‑ICD-Schockwendel (sendende Elektrode) zum S‑ICD-Gehäuse (empfangende Elektrode) übertragen und bilden so einen Kommunikationsvektor. Damit der LP das Signal empfängt, muss er sich im durch Wendel und Gehäuse definierten Feld befinden. Er erkennt die ATP-Anforderung, indem er die Spannungsdifferenz zwischen zwei Abtastelektroden misst, die sich am distalen und proximalen Ende des LP befinden. In der Studie von Tjong et al. erfolgte die Implantation des LP bei zwei Schafen über einen transfemoralen Zugang in den rechtsventrikulären Apex. Über einen zusätzlich eingebrachten Stimulationskatheter wurden ventrikuläre Tachykardien induziert. Der ATP-Befehl wurde manuell über das Programmiergerät des S‑ICD mittels Radiofrequenz-Kommunikation ausgelöst. Diese ATP-Anforderung wurde anschließend vom S‑ICD mittels unidirektionaler Kommunikation nach einem spezifischen Protokoll an den LP übertragen. Die Reaktion und Effektivität wurden ausgewertet, wobei die Autoren zeigen konnten, dass die erfolgreiche ATP-Abgabe zu 100 % (15/15 Versuchen) gelungen war. Die Erkennung des Kammerflimmerns durch den S‑ICD gelang auch dann, wenn durch provoziertes ventrikuläres Undersensing der LP eine VVI-Stimulation während des Kammerflimmerns durchgeführt hat.

Nach dieser Pilotstudie folgte die Validierung der Ergebnisse in einem größeren experimentell-prospektiven Tiermodell mit insgesamt 40 Tieren (Schafe, Schweine und Hunde; [16]). Das modulare System wurde sowohl im akuten als auch im chronischen Experiment über 90 Tage untersucht. Dabei wurden die Leistungsfähigkeit des LP, die Kommunikation vom S‑ICD zum LP, die Fähigkeit beider Systeme zur Rhythmusdetektion sowie die vom S‑ICD befohlene ATP-Therapie getestet.

**Abb. 3:**
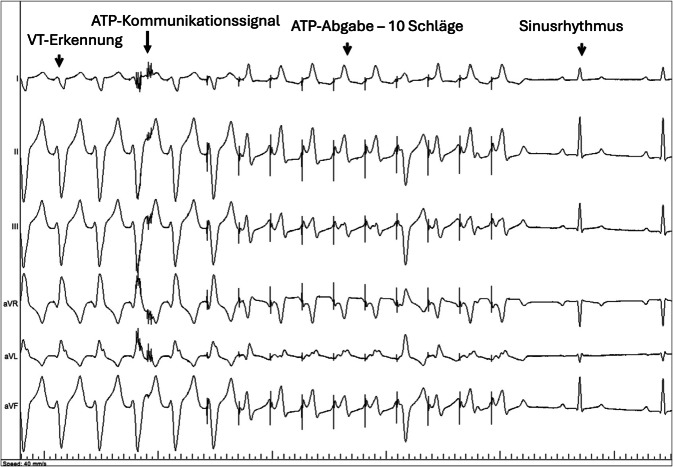
Beispiel einer induzierten monomorphen ventrikulären Tachykardie (VT), welche durch den subkutanen implantierbaren Kardioverter-Defibrillator (S-ICD) regelrecht detektiert wird und über eine unidirektionale Kommunikation zu einem ATP-Befehl vom S‑ICD zum Leadless Pacemaker (LP) führt. Nachfolgend kommt es zu einem ATP-Burst von 10 Schlägen mit 81 % der Zykluslänge, der die laufende VT erfolgreich terminiert. (Aus [16]; Lizenz: CC BY-NC-ND 4.0 (https://creativecommons.org/licenses/by-nc-nd/4.0/). Es wurden keine Änderungen vorgenommen)

Die Autoren zeigten, dass die kombinierte Implantation des LP und S‑ICD bei 98 % der Tiere erfolgreich war. Von diesen 39 Tieren wurden 23 über einen Zeitraum von 90 Tagen nach der Implantation nachbeobachtet. Die Funktion des LP war in allen untersuchten Fällen zufriedenstellend und zeigte ein stabiles VVI-Stimulationsverhalten über den gesamten Beobachtungszeitraum. Die unidirektionale Kommunikation vom S‑ICD zum LP war bei 99 % der Versuche erfolgreich (398 von 401), was in 100 % der Fälle zur Abgabe einer ATP-Therapie durch den LP führte (Abb. 3). Die korrekte Rhythmusdiskriminierung durch den S‑ICD konnte sowohl im Sinusrhythmus, unter ventrikulärer Stimulation durch den LP als auch bei ventrikulärer Tachykardie und Kammerflimmern beobachtet werden.

**Abb. 4:**
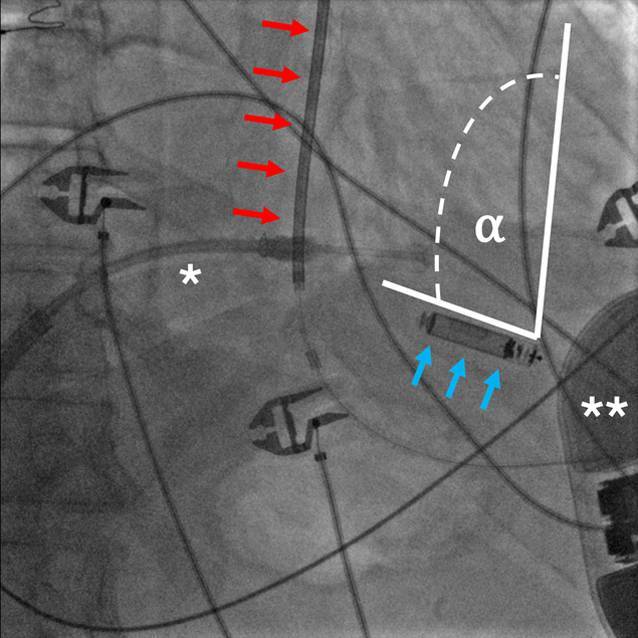
RAO-Projektion eines mCRM-Systems mit einem EMPOWER™ Leadless Pacemaker (LP) im rechtsventrikulären Apex und einem EMBLEM™ S‑ICD (subkutaner implantierbarer Kardioverter-Defibrillator) linksaxillär. Der Implantationswinkel ⍺ ist entscheidend für die Qualität der unidirektionalen Kommunikationsschwelle. Diese ist dann am besten, wenn die Achse des LP (*blaue Pfeile*) senkrecht zur S‑ICD-Schockwendel (*rote Pfeile*) ist und am schlechtesten, wenn der LP parallel zur S‑ICD-Schockwendel liegt. * Delivery Katheter, ** S‑ICD-Aggregat. (Bild mit freundlicher Genehmigung von Boston Scientific. © 2025 Boston Scientific Corporation oder verbundene Unternehmen. Alle Rechte vorbehalten)

Da sich der elektrische Kommunikationsvektor des S‑ICD zwischen der Schockwendel und dem Aggregat erstreckt und der LP diese Signale über seine Kathode und Anode aufnimmt, kann die räumliche Ausrichtung des LP in Bezug auf diesen Kommunikationsvektor die Fähigkeit des LP beeinflussen, die übertragenen Signale korrekt zu erfassen. In der Theorie wird das stärkste Signal vom LP dann wahrgenommen, wenn dessen Hauptachse in Richtung des Kommunikationsvektors des S‑ICD ausgerichtet ist (Abb. 4). Umgekehrt ist das Signal am schwächsten, wenn die Hauptachse des LP senkrecht zum S‑ICD-Vektor steht, oder wenn der LP parallel zur Schockwendel des S‑ICD liegt. Beim Menschen ist davon auszugehen, dass der LP in der Regel eine günstige Ausrichtung zum Kommunikationsvektor des S‑ICD hat, insbesondere bei einer Implantation im apikoseptalen Bereich des rechten Ventrikels, der als bevorzugte Position gilt. Eine Platzierung des LP in der freien Wand des rechten Ventrikels wird hingegen nicht empfohlen, da dort sowohl die Ausrichtung für die Signalübertragung ungünstiger ist als auch ein erhöhtes Risiko für eine myokardiale Perforation besteht [12].

Die Langzeitergebnisse der bislang größten präklinischen Studie wurden von Breeman et al. veröffentlicht [1]. In dieser Studie wurde neben der elektrischen Performance und der Implantationssicherheit des LP auch die Explantierbarkeit des mCRM-Systems bei 68 Hunden über einen Zeitraum von bis zu 18 Monaten untersucht. Als Leistungsparameter wurde u. a. die Kommunikationsschwelle zwischen dem S‑ICD und dem LP untersucht. Diese war definiert als die minimale Signalspannung, bei der eine ATP-Anforderung vom LP bei zwei aufeinanderfolgenden Versuchen erfolgreich empfangen wurde. Die Kommunikationsschwelle war abhängig von der Orientierung des LP relativ zur Schockwendel des S‑ICD und blieb im Durchschnitt sowohl akut-perioperativ als auch nach 18 Monaten unterhalb der nominalen Einstellung und dem minimal programmierbaren Schwellenwert. Die Stimulationsreizschwelle des LP stieg zunächst leicht an, blieb anschließend aber konstant; Impedanz und R‑Wellen-Amplituden zeigten einen moderaten, aber klinisch irrelevanten Rückgang. Einflussfaktoren wie Rückenlage oder ein größerer Winkel zwischen dem LP und der S‑ICD-Sonde zeigten in der multivariablen Analyse lediglich geringe Effekte auf die Kommunikationsschwelle. Insgesamt sprechen die Daten für eine hohe technische Zuverlässigkeit und elektrische Langzeitstabilität des modularen Systems.

**Abb. 5:**
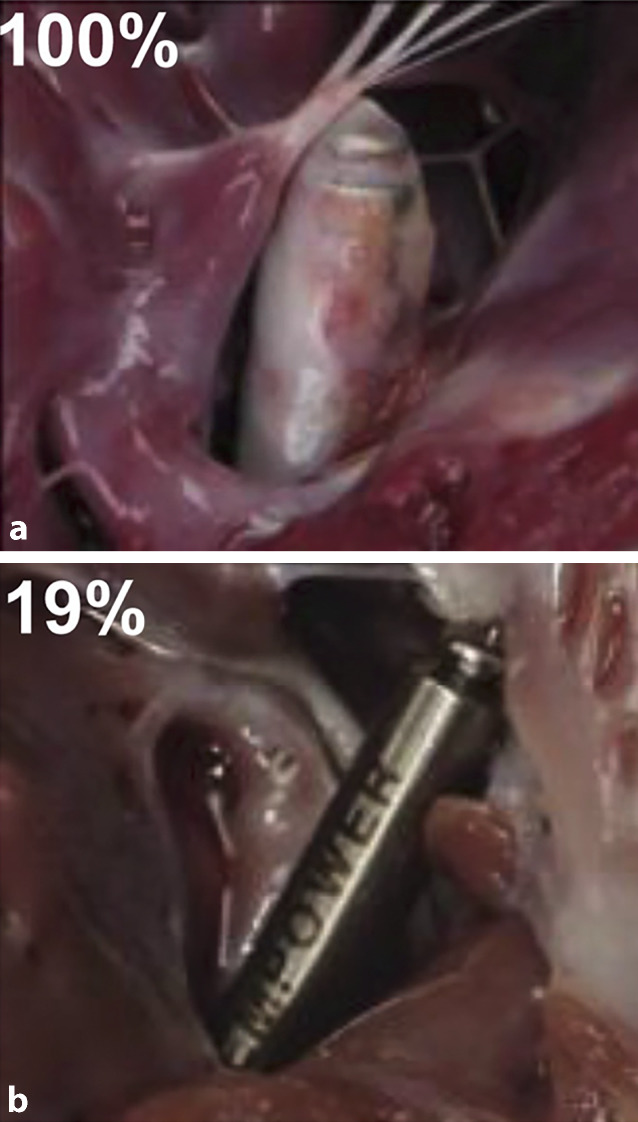
Verschiedene Stadien der Einheilung mit unterschiedlichem Ausmaß an fibröser Kapselbildung des Leadless Pacemaker (LP), gezeigt anhand Nekropsiestudien am Tag 91 (**a**) und Tag 540 (**b**). (Aus [1]. © 2022 The Authors. Veröffentlicht unter der Creative Commons Lizenz CC BY 4.0 (https://creativecommons.org/licenses/by/4.0/). Es wurden keine Änderungen vorgenommen)

Bezogen auf die Sicherheit des mCRM-Systems wurden nur wenige Komplikationen beobachtet, die größtenteils auf Prototypkomponenten oder anatomische Besonderheiten des Modells zurückzuführen waren. Weder hämodynamisch relevante Klappeninsuffizienzen noch Perikardergüsse wurden beobachtet. Schließlich wurden auch verschiedene Strategien zum Austausch des mCRM-Systems getestet, einschließlich Bergung, Neuimplantation nach Bergung und paralleler Implantation weiterer LP. Eine Explantation war in 17 von 28 Fällen erfolgreich; die Hauptursachen für Misserfolge waren vollständige Verkapselung oder Gewebeeinwuchs in die Fixationsstrukturen (Abb. 5). Die elektrische Funktion von neu implantierten LP, auch bei gleichzeitiger Anwesenheit von bis zu drei Geräten, blieb stabil. Eine relevante mechanische oder elektrische Beeinträchtigung durch mehrere LP wurde nicht beobachtet, mit Ausnahme eines einzelnen Falls erhöhter Stimulationsschwelle. Diese Ergebnisse legen nahe, dass sowohl Explantation als auch „Abandonment-Strategien“ in der klinischen Praxis machbar sind, wobei die optimale Herangehensweise noch weiter untersucht werden muss.

## Klinische Evidenz: MODULAR-ATP-Studie – Design, Population und Hauptendpunkte

Eine Vielzahl prospektiver Studien hat inzwischen die Sicherheit und Wirksamkeit des S‑ICD belegt [5, 8]. Zu den besonderen Vorteilen zählen die längere Lebensdauer der Elektroden und das Fehlen von transvenösen Elektroden, wodurch das Risiko einer Sondeninfektion sowie struktureller Schäden an Venen und Herzklappen entfällt. Trotz verbesserter Detektionsalgorithmen und damit deutlich reduziertem Risiko inadäquater Schocks bestehen nach wie vor technische Limitationen [2, 14]. Besonders relevant ist hierbei die fehlende Möglichkeit der antitachykarden Therapie monomorpher ventrikulärer Tachykardien sowie der längerfristigen antibradykarden Stimulation bei intermittierender Bradykardie. Mit der Möglichkeit einer modularen Integration des EMPOWER™ LP entweder zur De-novo-Implantation oder zu einem bereits vorbestehenden EMBLEM™ S‑ICD könnte diese technische Limitation behoben werden.

Die MODULAR-ATP-Studie (NCT04798768) wurde als globale, multizentrische, Single-arm-Studie konzipiert, um die Sicherheit und Wirksamkeit des mCRM-Systems zu evaluieren. Die Studie schließt prospektiv nichtrandomisiert bis zu 300 Patienten, mit einer Klasse-I-, Klasse-IIa- und Klasse-IIb-Indikation für einen ICD und einem erhöhten Risiko für zukünftige monomorphe ventrikuläre Tachykardien ein. Die primären Studienendpunkte sind: Erfolgsraten der Implantation, ATP-Erfolgsrate bei induzierten und spontanen Tachyarrhythmien sowie die Sicherheit und das Auftreten schwerer Nebenwirkungen während eines definierten Nachsorgezeitraums von mindestens 12 Monaten [10]. Als Sicherheitsendpunkt wurde die Freiheit von schwerwiegenden Komplikationen innerhalb der ersten 6 Monate nach Implantation von > 86 % definiert. Schwerwiegende Komplikationen wurden definiert als alle unerwünschten Ereignisse im Zusammenhang mit dem LP oder dessen Implantationsverfahren, die eine Systemrevision, einen dauerhaften Funktionsverlust des LP, eine Hospitalisierung oder den Tod zur Folge haben. Die Patienten werden regelmäßig bis zu 5 Jahre nachverfolgt.

## Initialergebnisse der MODULAR-ATP-Studie: Sicherheit, Effektivität und Pacing-Performance

In die MODULAR-ATP-Studie wurden 293 Patienten an 38 Zentren eingeschlossen [7]. Davon haben 162 Patienten die 6‑Monats-Visite erreicht. Das mittlere Alter war 60 Jahre mit 83 % männlichen Studienteilnehmern. Die primärprophylaktische ICD-Indikation bestand bei der knappen Mehrheit (54 %), mit am häufigsten vorkommender ischämischer Kardiomyopathie als Grunderkrankung (61 %). Bei allen Studienteilnehmern konnte ein mCRM-System implantiert werden. Dabei wurde bei 41 % isoliert ein zusätzlicher LP zum bestehenden S‑ICD implantiert. 96 (59 %) Patienten erhielten den LP und S‑ICD im selben operativen Eingriff. Die mediane Prozedurdauer betrug 35 min für die LP-Implantation und rund 79 min für den kombinierten LP/S-ICD-Eingriff. Intraprozedurale Repositionierung des LP war bei 43 Patienten (27 %) erforderlich. Bei fast allen Patienten (94 %) wurde eine Defibrillationstestung durchgeführt. Die Terminierung des Kammerflimmerns mit dem ersten Schock war bei 94 % der Patienten möglich.

Die mediane Nachsorge betrug 12,4 Monate und 151 Patienten erreichten die 6‑Monats-Visite. Die erfolgreiche Kommunikation zwischen dem S‑ICD und LP wurde bei 98,8 % aller Kommunikationstests beobachtet. Damit wurde das vordefinierte Performanceziel von 88 % übertroffen. Die mediane Reizschwelle des LP betrug 0,7 V bei Implantation und 0,56 nach 6 Monaten. Die Amplitude der R‑Zacke betrug initial 11,1 und 14,6 mV im Verlauf. Damit wurde auch der zweite Performanceendpunkt von mindestens 80 % überboten.

Die ATP-Therapie wurde aufgrund von 31 spontanen Arrhythmie-Episoden bei insgesamt 13 Patienten abgegeben und terminierte 61 % der Episoden. Alle Episoden, die nicht durch ATP beendet wurden, terminierten entweder spontan oder wurden durch den S‑ICD terminiert. Adäquate Therapie (ATP oder Schock) wurde bei insgesamt 9 % der Patienten abgegeben. Inadäquate Therapie trat bei 14 Patienten auf und war mehrheitlich verursacht durch Oversensing langsamer ventrikulärer Tachykardie. Dabei kam es bei 5 % zu einem inadäquaten Schock. Keine inadäquaten Episoden wurden hingegen aufgrund eines Oversensing der Stimulation durch den LP beobachtet. Außerdem wurden keine Fälle beobachtet, in denen eine fehlerhafte Kommunikation zwischen dem LP und dem S‑ICD zu einem Ausbleiben der Therapieantwort geführt hätte.

Die Ergebnisse der MODULAR-ATP-Studie waren vielversprechend. Es wurden Implantationserfolgsraten von über 98 % erzielt. Die erfolgreiche ATP-Therapie erwies sich mit rund 61 % als vergleichbar effektiv wie bei anderen historischen ICD-Studien, sowohl mit transvenösen als auch dem extravaskulären ICD-System [4, 17]. Die stabile drahtlose Kommunikation zwischen dem EMPOWER™-Pacemaker und dem EMBLEM™ S‑ICD wurde klinisch bestätigt und zeigte eine nahezu fehlerfreie Leistung über den Studienzeitraum.

Elektrische Parameter, wie Reizschwellen und Wahrnehmungsamplituden, blieben stabil. Nebenwirkungen und Komplikationen waren selten und lagen unterhalb der historisch dokumentierten Raten für transvenöse Systeme, was das günstige Sicherheitsprofil bestätigt.

## Stellenwert des ATP in der gegenwärtigen ICD-Therapie

Seit Veröffentlichung der MADIT-RIT-Studie ist bekannt, dass eine verzögerte ICD-Intervention die Prognose der Patienten verbessert und mit einer verringerten Mortalität assoziiert ist [11]. Die heutige ICD-Programmierung zielt darauf ab, Schockabgaben möglichst zu vermeiden. Dies wird u. a. erreicht durch die Verhinderung inadäquater Therapien, die Vermeidung unnötiger Interventionen bei selbstlimitierenden ventrikulären Arrhythmien – z. B. durch Verzögerung des Therapiebeginns und die Auswahl geeigneter Interventionszonen, um durch die optimierte Nutzung das Maximum an Effektivität der ATP zu erzielen. Die Einführung des S‑ICD, der potenzielle Komplikationen endovaskulärer Systeme vermeidet, jedoch nicht über ATP-Funktionalität verfügt, unterstreicht die Notwendigkeit, geeignete Subgruppen innerhalb der Präventionskohorten zu identifizieren, für die entweder subkutane oder endovaskuläre Systeme den größten klinischen Nutzen bieten. In der PRAETORIAN-Studie konnten 46 % der ventrikulären Tachykardie-Episoden im transvenösen Arm erfolgreich durch ATP beendet werden. Allerdings kam es nur bei 54/423 Patienten (13 %) zu einer adäquaten ATP-Therapie innerhalb von 5 Jahren [8].

In der PARTITA-Studie wurden insgesamt 517 Patienten mit ischämischen und nichtischämischen Kardiomyopathien untersucht, die ein ICD-System erhielten. Von diesen entwickelten 154 Patienten (30 %) eine anhaltende ventrikuläre Tachykardie (VT), und lediglich 112 Patienten (22 %) erhielten eine ATP-Therapie im Rahmen einer modernen, leitliniengerechten Geräteprogrammierung [3].

Schließlich zeigte die APPRAISE-ATP-Studie eine Überlegenheit der ATP-Therapie mit einer relativen Risikoreduktion von 28 % in Bezug auf die Zeit bis zum ersten Schock jeglicher Ursache – im Vergleich zwischen der ATP-aktivierten und der ATP-deaktivierten Gruppe (Log-Rank-*p*-Wert = 0,005) [13]. Dies entspricht einer absoluten Reduktion aller Schockereignisse um etwa 1 % pro Jahr bei Patienten mit einer primärpräventiven ICD-Indikation. Interessant war, dass sich die Gesamtzahl der Schockabgaben zwischen den beiden Studiengruppen nicht signifikant unterschied (*p* = 0,38). Zudem bestand ein höheres Risiko für einen VT/VF-Sturm (HR: 2,39; *p* = 0,006) und eine numerisch höhere Anzahl an Todesfällen in der ATP-aktivierten Gruppe (HR: 1,15; *p* = 0,184). Dies verdeutlicht, dass es keine Hinweise auf eine erhöhte Mortalität in der ATP-deaktivierten Gruppe gab.

## Klinische Implikationen und zukünftige Perspektiven

Die Ergebnisse der MODULAR-ATP-Studie legen nahe, dass das mCRM-System erhebliche klinische Vorteile bietet und eine breite Anwendung in der klinischen Praxis erfahren könnte.

Das zukünftige Potenzial eines modularen Systems, bei dem ein S‑ICD bidirektional mit einem LP oder anderen implantierten Modulen kommuniziert, ist vielversprechend. Die direkte Übertragung von Informationen zu Rhythmus und Herzfrequenz zwischen dem LP und dem S‑ICD könnte inadäquate Therapien durch T‑Wellen-Oversense reduzieren. Da bei diesem System das Herz und die Gefäße nichtinvasiv beansprucht werden, könnten gleichzeitig Schrittmacher- und Defibrillator-Funktionen bereitgestellt werden, ohne intravaskuläre und intrakardiale Strukturen zu kompromittieren.

Ein wesentlicher Vorteil dieses modularen Ansatzes liegt darin, dass derzeit nicht zuverlässig vorhergesagt werden kann, welcher Schrittmacherpatient später eine Defibrillatortherapie benötigen wird oder umgekehrt. Diese Unsicherheit erschwert heute noch die klinische Akzeptanz der jeweiligen Einzelgeräte. Ein modular aufgebautes System könnte die Implantation unnötiger invasiver Komponenten vermeiden, da Patienten zunächst nur das Gerät erhalten, für das eine aktuelle Indikation besteht. Sollten später zusätzliche Therapieoptionen erforderlich werden, könnte das System individuell erweitert und an den Patienten angepasst werden.

Darüber hinaus ist es angesichts der Fortschritte im Bereich der kabellosen Zweikammer-Herzschrittmacher durchaus denkbar, dass zukünftige mCRM-Systeme auch die kardiale Resynchronisationstherapie (CRT) umfassen könnten.

## Fazit für die Praxis

- Die Kombination eines subkutanen Defibrillators (S-ICD) mit dem EMPOWER™ Leadless Pacemaker (LP) zur antibradykarden Stimulation und Überstimulation (antitachykarde Stimulation, ATP) stellt im Sinne eines modularen Therapieansatzes eine zukunftsweisende Erweiterung der extravaskulären Devicetherapie dar.
- Sie bietet insbesondere für Patientinnen und Patienten mit bestehender Indikation zur Implantation eines S‑ICD eine Option zur simultanen oder späteren bedarfsorientierten, modularen Erweiterung bei Entwicklung einer Bradykardie oder Indikation zur ATP.
- Einschränkend ist zu berücksichtigen, dass zum gegenwärtigen Zeitpunkt keine Möglichkeit zur kabellosen Mehrkammer- oder resynchronisierenden Stimulation (z. B. kardiale Resynchronisationstherapie [CRT] oder Conduction System Pacing [CSP]) besteht. Entsprechende Systeme wären perspektivisch wünschenswert.

## Data Availability

Nicht zutreffend
